# Avian Influenza Virus with Hemagglutinin-Neuraminidase Combination H8N8, Isolated in Russia

**DOI:** 10.1128/genomeA.00545-14

**Published:** 2014-06-05

**Authors:** Mariya V. Sivay, Kirill A. Sharshov, Mary Pantin-Jackwood, Vladimir V. Muzyka, Alexander M. Shestopalov

**Affiliations:** a FGBI Scientific Center of Clinical and Experimental Medicine (SB RAMS), Novosibirsk, Novosibirsk Region, Russia; b Novosibirsk State University, Novosibirsk, Novosibirsk Region, Russia; c Southeast Poultry Research Laboratory, USDA, Athens, Georgia, USA

## Abstract

We report the genome sequence of an avian influenza virus (AIV) subtype H8N8, isolated in Russia. The genome analysis shows that all genes belong to AIV Eurasian lineages. The PB2 gene was similar to a Mongolian low-pathogenic (LP) AIV H7N1 and a Chinese high-pathogenic (HP) AIV H5N2.

## GENOME ANNOUNCEMENT

Surveillance for avian influenza virus (AIV), *Orthomyxoviridae* family, in wild birds all over the world has increased the data available on virus subtypes. Most of the viruses have been detected in wild waterfowl and poultry, but the virus has also been isolated from several mammal species, including humans (1). More than 140 hemagglutinin-neuraminidase (HA-NA) subtype combinations have been detected (2–4). AIV H8 subtypes have been isolated in both hemispheres, but most of the viruses were detected in North America. There is information about 147 virus strains of the H8 subtype in the Influenza Virus Resource (5). The most common subtype combination is H8N4, but the H8 subtype was also detected with N2, N3, N5, and N7 NA subtypes. All the H8 subtype viruses were isolated from wild ducks and turkeys. The information from the AIV H8N8 subtype isolation was mentioned by Abenes (6) and Daum et al. (7), but genome sequence data are not available.

This surveillance study was conducted in Western Siberia, the Asian part of Russia. The territory crosses by the Central Asian flyway, which is used by combined bird populations of Europe, Asia, and Africa (8). During fall 2005 to spring 2006 in Western Siberia, a high-pathogenic (HP) AIV H5N1 subtype outbreak was detected; numerous wild water birds and poultry were infected. Later, the HP virus was also detected in several European countries. Biological and phylogenetic studies showed that the HP AIV H5N1 was related to the Chinese HP H5N1 (clade 2.2), which caused a serious outbreak in bar-headed goose and cormorant populations in China, in spring 2005 (9).

In this study, the H8N8 AIV strain was isolated from a common teal (*Anas crecca*) in Russia (Chany Lake) in fall 2009. Molecular, biological, and phylogenetic analyses of A/teal/Chany/444/2009(H8N8) were conducted. The HA protein has no multibasic cleavage site. The pathogenicity of the virus was determined in chickens. None of the chickens showed any clinical sings of disease. The intravenous pathogenicity index (IVPI) is 0.

Phylogenetic analysis of all eight genes shows that A/teal/Chany/444/2009(H8N8) is close to the Eurasian AIV lineages. The HA gene is similar to that of A/mallard/Netherlands/1/2006(H8N4), with 97% identity. The NA gene is most closely related to that of A/mallard/Sweden/45/2002(H11N8), with identity of 98%. The PB2 gene is closely related to those of A/duck/Mongolia/867/2002(H7N1) (LP) and A/duck/Hebei/0908/2009(H5N2) (HP), with identity levels of 98% and 97%, respectively. The PB1 and PA genes are similar to those of A/white-fronted goose/Mongolia/1-125/2008(H3N8) (99% identity). The NP, M, and NS genes are most similar to those of A/northern shoveler/Mongolia/907V/2009(H10N8) (99% identity), A/duck/Korea/DY104/2007(H4N6) (99% identity), and A/duck/Italy/194659/2006(H3N2) (100% identity).

Analysis of the M2 amino acid sequence of the A/teal/Chany/444/2009(H8N8) virus indicates that it possesses the 55F substitution, an enhanced transmission phenotype marker (10). Amino acid analysis of the M protein also shows no substitutions related to drug resistance in the A/teal/Chany/444/2009(H8N8) strain.

The isolation of the rare AIV H8N8 subtype shows the importance of surveillance for viruses in their natural reservoir and the significance of Western Siberia in virus ecology, persistence, and evolution.

### Nucleotide sequence accession numbers.

The H8N8 whole genome sequence has been deposited in GenBank under the accession numbers CY098521 to CY098528.
